# Prominent Lymphovascular Space Invasion as an Independent Prognostic Factor in LVSI-Positive Endometrioid Endometrial Carcinoma: Evidence for a Novel Three-Tiered Classification

**DOI:** 10.3390/jcm15093278

**Published:** 2026-04-25

**Authors:** Tuğçe Sırma, Goncha Kamallı, Gürdeniz Serin, Göktuğ Aydoğan, Osman Zekioğlu, Senem Alanyalı, Zeynep Özsaran, Erdem Göker, Ahmet Aydın Özsaran, Mustafa Coşan Terek, Levent Akman, Nuri Yıldırım

**Affiliations:** 1 Department of Obstetrics and Gynecology, Division of Gynecological Oncology, İskenderun State Hospital, Hatay 31200, Türkiye; drtugcesirma@hotmail.com; 2 Department of Obstetrics and Gynecology, Medigün Hospital, Manisa 45500, Türkiye; goncakamal@yahoo.com; 3 Department of Pathology, Faculty of Medicine, Ege University, İzmir 35100, Türkiye; drgurdeniz@gmail.com (G.S.); aydogangoktug10@gmail.com (G.A.); osman.zekioglu@ege.edu.tr (O.Z.); 4 Department of Radiation Oncology, Faculty of Medicine, Ege University, İzmir 35100, Türkiye; senem.alanyali@gmail.com (S.A.); zeynep.ozsaran@ege.edu.tr (Z.Ö.); 5 Department of Medical Oncology, Faculty of Medicine, Ege University, İzmir 35100, Türkiye; erdem.goker@ege.edu.tr; 6 Department of Obstetrics and Gynecology, Division of Gynecological Oncology, Faculty of Medicine, Ege University, İzmir 35100, Türkiye; ozsarana@gmail.com (A.A.Ö.); terekmc@yahoo.com (M.C.T.); leventakman@gmail.com (L.A.)

**Keywords:** survival, recurrence, lymphovascular space invasion, endometrioid endometrial cancer

## Abstract

**Background/Objectives**: Lymphovascular space invasion (LVSI) is an established prognostic factor in endometrial cancer, yet current risk stratification relies on a binary focal/substantial classification. Whether further substratification into a “prominent” LVSI category (≥10 involved vessels) provides independent prognostic value beyond the FIGO 2023 staging framework remains unclear. We aimed to investigate the prognostic significance and clinical outcomes regarding the extent of lymphovascular space invasion (LVSI) in patients with LVSI-positive endometrioid endometrial cancer (EEC). **Methods**: We retrospectively identified 94 patients with International Federation of Gynecology and Obstetrics (FIGO) 2009 stage I–IV EEC who underwent surgical staging. LVSI-positive cases were classified as focal (1–4 involved vessels), substantial (5–9 involved vessels), or prominent (≥10 involved vessels). **Results**: Of the 94 LVSI-positive patients, 58 (61.7%) had focal, 11 (11.7%) had substantial, and 25 (26.6%) had prominent LVSI. Five-year overall survival (OS) rates were 95.3%, 72.7%, and 53.0% for focal, substantial, and prominent LVSI, respectively (*p* < 0.001), with corresponding progression-free survival rates of 87.6%, 71.6%, and 34.3% (*p* < 0.001). On multivariable analysis, prominent LVSI emerged as an independent predictor of both mortality (HR 14.31, *p* < 0.001) and disease progression (HR 4.02, *p* = 0.009), retaining significance after re-assessment for FIGO 2023 stage. Tumor size > 4 cm and p53-abnormal status were also independently associated with OS. **Conclusions**: Prominent LVSI was independently associated with aggressive disease and poor survival in EEC; recognizing its extent as a prognostic parameter may improve both clinical management and future risk classification frameworks. As these findings are derived from a single-institution, LVSI-positive cohort with an empirically defined threshold, external validation is warranted before clinical adoption.

## 1. Introduction

Endometrial cancer (EC) is the most prevalent neoplasm in the genital tract in developed countries, with a relatively favorable prognosis, but approximately 2% to 10% of the International Federation of Obstetrics and Gynecology (FIGO) 2009 stage I cases recur [1,2]. The standard approach of EC is surgery including total hysterectomy, bilateral salpingo-oophorectomy (BSO), and lymph node (LN) assessment—including selective pelvic ± para-aortic lymph node dissection or sentinel lymph node biopsy in eligible patients—with administration of adjuvant therapy in selected cases [3,4]. The surgical specimens obtained from the hysterectomy-based surgical staging procedure are of significant value in identifying histological factors that are conducive to the determination of the patient’s prognosis [5]. A prime example of such factors is lymphovascular space invasion (LVSI) [5]. LVSI has been demonstrated to be associated with high-risk features in EC, including deep myometrial invasion (MI) and high-grade histologies [6]. LVSI is present in approximately 8–10% of FIGO stage I EC patients [7], and has been associated with a higher risk of LN metastasis, pelvic/distant recurrence, and poor overall survival (OS) [8].

The most common method of grading the presence of LVSI in hysterectomy specimens is by employing a two-tiered scoring system (absence vs. presence) [9]. Hachisuga and colleagues [10,11,12] classified LVSI in a three-tiered scoring system (none, mild and severe) and concluded that this scoring was closely related to LN involvement and prognosis. Fujimoto and colleagues [13] adopted a different categorization of LVSI, classifying it as nil (-), mild (+), moderate (++) or prominent (+++). Their findings indicated that patients within the moderate/prominent group exhibited a significantly higher prevalence of positive pelvic LNs [11]. A pooled analysis of Post-Operative Radiation Therapy in Endometrial Carcinoma (PORTEC) 1 and 2 examined various LVSI scoring systems and demonstrated that a three-tiered scoring system (none, focal, or substantial) predicted pelvic regional recurrence, distant metastasis, and OS, that patients with substantial LVSI potentially benefited from external beam radiation therapy (EBRT) [14], and that the four-tiered approach had no added value over the latter [11]. Pifer and colleagues [14] conducted a study of surgically staged patients with node-negative stage I endometrioid endometrial cancer (EEC). The study utilized a cutoff of four or more vessels involved on at least one pathology slide to define substantial LVSI and found no significant correlation between LVSI and pattern of recurrence [6,14]. Peters and colleagues [15] established a consensus on the definition of substantial LVSI, which is characterized by the presence of four or more vessels on a single slide. This is in contrast to the World Health Organization (WHO) 2020 definition of substantial LVSI, which is defined as involvement of five or more vessels on at least one pathology slide [16]. In 2023, FIGO recently updated the EC staging system according to the WHO definition [17]. The European Society of Gynaecological Oncology (ESGO), the European Society of Radiotherapy and Oncology (ESTRO), and the European Society of Pathology (ESP) 2025 guidelines describe the clinical approach and management of patients with EC and state that substantial LVSI is one of the risk factors that categorizes patients as a high–intermediate-risk group who should receive adjuvant EBRT for optimal pelvic control [18]. In contrast, the National Comprehensive Cancer Network (NCCN) 2025 Clinical Practice Guidelines define substantial LVSI as involvement of four or more vessels while continuing to base treatment recommendations on the FIGO 2009 staging system [19]. While the binary focal/substantial classification has proven clinically useful, we hypothesized that further stratification of cases with extensive LVSI might identify a subgroup with particularly aggressive tumor biology. The threshold of ten or more vessels was selected based on preliminary observations of outcome clustering in our cohort and represents approximately twice the WHO threshold for substantial LVSI, potentially capturing cases with exceptionally high tumor embolic burden. This threshold therefore requires independent external validation before it can be incorporated into routine pathological reporting.

In light of these processes, the objective of the present study was to investigate the prognostic role of the different LVSI grading in patients with EEC.

## 2. Materials and Methods

### 2.1. Study Design

The study protocol was approved by Ege University Medical Research Ethics Committee (approval date: 7 March 2025; decision no: 24-3T/49) based on the ethical standards described in the Declaration of Helsinki. Patients with EEC who underwent surgical staging for primary treatment from July 2005 to December 2021 at the Department of Gynecological Oncology of Ege University, İzmir, were reviewed retrospectively. The medical records were obtained from the hospital’s electronic database and patient files. We collected their clinicopathological information, including age, menopausal status, surgery details, need for adjuvant treatment, recurrence and survival outcomes.

This study included patients with EEC with histopathologically confirmed LVSI in the hysterectomy specimen. Cases with non-endometrioid or mixed-type histology, endometrial hyperplasia, or no LN assessment at the time of surgery were not included, in addition to those with incomplete medical records. Patients were also excluded from the study if they had synchronous or metastatic cancer, or received neoadjuvant chemotherapy (NACT) or radiation.

### 2.2. Clinical Information

Preoperative clinical evaluation consisted of general and gynecological examination, performance status, blood tests, serum Cancer Antigen 125 (CA-125) levels, electrocardiogram (ECG), and transvaginal ultrasonography (TVUSG). Positron emission tomography/computed tomography (PET-CT) or pelvic magnetic resonance imaging (MRI) was performed in patients with suspected extrauterine disease based on clinical examination, elevated CA-125 levels exceeding 35 U/mL, or equivocal ultrasound findings. During the preoperative evaluation, patients with clinically uterine-confined EEC were selected for surgical intervention. All patients underwent surgical staging consisting of total hysterectomy with BSO, pelvic washing and LN assessment. Firstly, pelvic washing for cytology (using approximately 50 mL of normal saline) was performed. Where necessary, suspicious lesions and LNs were sampled. Selective bilateral pelvic ± para-aortic lymph node dissection (BPPALND) and omental biopsy were performed based on patient risk of LN metastases according to the Mayo criteria and frozen section analysis: grade 3 tumor, MI ≥ 50%, cervical stromal invasion and grade 2+tumor size > 2 cm. Gynecologic oncologists performed each procedure with the goal of obtaining thorough surgical staging by laparoscopy (L/S) or laparotomy (L/T) [20]. The uterine manipulator was utilized in all laparoscopy patients [21]. The pelvic lymph node dissection (PLND) entailed the bilateral excision of level 1 and 2 nodes [21]. The para-aortic lymph node dissection (PALND) comprised the excision of level 3 nodes [21].

All histologic examinations including grade, tumor size (<4 cm or ≥4 cm), location and cervical–isthmic involvement, the depth of MI, LN status and adnexal involvement of the tumor were conducted by pathologists specialized in gynecological oncology [21]. The FIGO 2009 staging system was utilized for the staging of the cases [20]. For patients treated prior to 2009, stage was retrospectively determined based on surgical and pathological assessment [20].

The decision regarding the administration of adjuvant treatment was deliberated at a multidisciplinary tumor board. Treatment recommendations were generally aligned with the ESGO-ESTRO-ESP guidelines contemporaneously in effect at the time of each patient’s treatment (i.e., the 2016 guidelines for patients treated before 2021, and the 2020/updated recommendations thereafter), and individualized according to prognostic factors including tumor size, grade, molecular status, age, LN status, MI, and FIGO stage. Because the study spans 2005–2021, minor variations in practice reflect the evolution of guideline recommendations over this period. The patients received one of the following adjuvant treatment modalities: brachytherapy alone, EBRT alone, combined brachytherapy and EBRT, or combined radiotherapy and platinum-based chemotherapy (sequential or concurrent chemoradiation). The latter was generally reserved for patients with high-risk or advanced-stage disease (FIGO 2009 stage III–IV) as per multidisciplinary board recommendation. Treatment was dependent on a combination of risk factors, including patient age, tumor grade, and depth of MI. Patients with advanced-stage disease, defined as FIGO 2009 stage III or IV, received adjuvant chemotherapy (CT). The preferred adjuvant chemotherapy combination was paclitaxel and carboplatin. The period of time between the surgical procedure and commencement of radiotherapy (RT) was 6–8 weeks.

The patients were monitored on a three-monthly basis for a period of two years, following which they were observed on a six-monthly basis for a further three years, and then on an annual basis thereafter. Follow-up visits were structured according to institutional protocol, consistent with ESMO, NCCN and ESGO guidelines in effect during the study period, and comprised physical and pelvic examination, vaginal cytology, serum CA-125 measurement, and chest radiography. Chest radiography was performed at each follow-up visit as a first-line screening tool to detect pulmonary metastasis; cross-sectional imaging (CT or PET-CT) was subsequently obtained when radiographic findings raised concern for recurrent disease. Locoregional relapses were categorized according to their site of recurrence, namely, pure vaginal, pelvic non-vaginal, and isolated pelvic nodal recurrence and distant relapse isolated para-aortic nodal, or distant recurrence [1]. The term multifocal spread is defined as patients with both local and distant recurrence [1]. Progression-free survival (PFS) was calculated from the date of surgery or end of adjuvant treatment to the earliest date of progression, recurrence of disease or death due to disease. OS was calculated from the date of surgery to December 2023 or the date of death. The close date for this study was 31 December 2023.

### 2.3. Definition of LVSI and Scoring System

**Figure 1:**
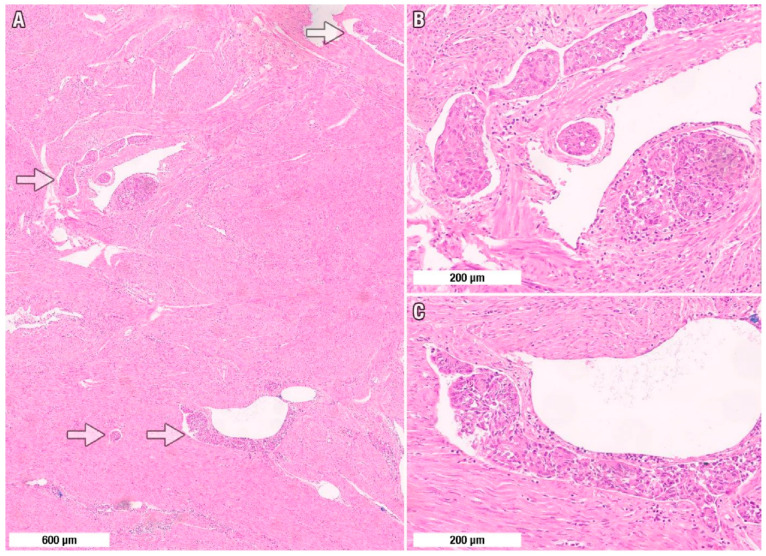
Spectrum of morphologic appearances of lymphovascular space invasion (LVSI) in endometrioid endometrial carcinoma. (**A**) Low-power hematoxylin and eosin view demonstrating multifocal LVSI between myometrial smooth-muscle bundles (arrows). (**B**,**C**) High-power views show cohesive intraluminal tumor emboli molded to thin-walled lymphatic/small venous channels and apposed to an intact endothelial lining—features that support true LVSI rather than stromal retraction artifact. Scale bars: (**A**), 600 μm; (**B**–**C**), 200 μm.

**Figure 2:**
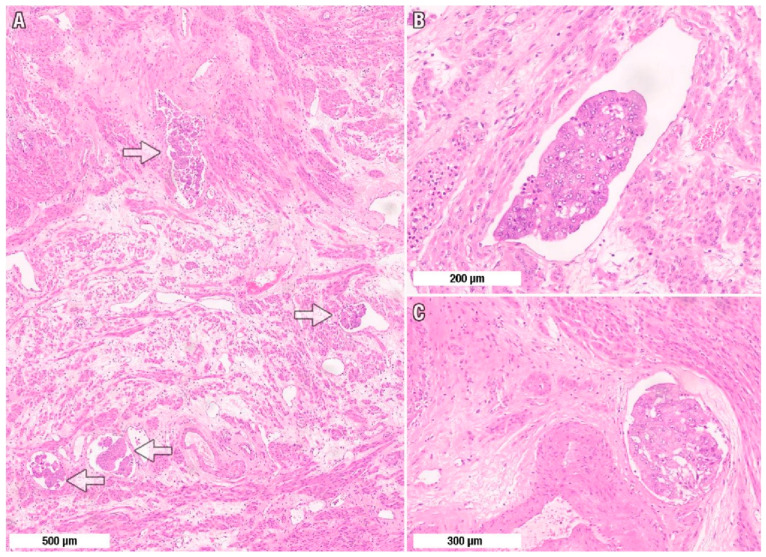
Representative hematoxylin and eosin images of lymphovascular space invasion (LVSI) in endometrioid endometrial carcinoma. (**A**) Prominent LVSI: cohesive carcinoma nests within endothelial-lined lymphovascular channels (arrows). (**B**,**C**) Higher-power views show intraluminal tumor groups conforming to the vessel contour—(**B**) elongated channel and (**C**) round-caliber vessel with a tumor plug. Scale bars: (**A**), 500 μm; (**B**), 200 μm; (**C**), 300 μm.

The presence of LVSI is defined as the unequivocal presence of non-necrotic tumor cells or tumor cell clusters within an endothelial-lined space in either the lymphatic vessels or vascular vessels (Figure 1) [21]. In cases where a tumor was positive for LVSI, the hematoxylin and eosin (H&E)-stained slides were independently re-reviewed by two gynecologic pathologists (G.S. and O.Z.), who were blinded to each other’s assessments. Initial independent assessment by two gynecologic pathologists yielded concordant LVSI classification in 91 of 94 cases (96.8% agreement). The three discordant cases were resolved through consensus review with a third pathologist using a multiheaded microscope. The extent of LVSI was classified as follows: focal, substantial or prominent (Figure 2) [10,21,22].

(1) Focal: 1–4 involved vessels on at least one slide;
(2) Substantial: 5–9 involved vessels on at least one slide;
(3) Prominent: 10 or more involved vessels on at least one slide.

Cases initially reported as negative for LVSI on the pathology report were not subjected to further review [23]. Intratumoral LVSI was not considered [7]. In the event of potential mimics, such as retraction/shear artifacts, smear artifacts and microcystic, elongated and fragmented (MELF) patterns, the designation of involved foci as LVSI was deemed to be unfeasible [7].

### 2.4. Outcomes

The main endpoint of the current study was to examine survival outcomes and recurrence in accordance with extent of the LVSI status. The principal secondary efficacy endpoint of the research was to explore prognostic factors that potentially correlated with the extent of LVSI.

### 2.5. Statistical Analysis

The data was analyzed and evaluated using the SPSS 26.0 software package (IBM Corp., Chicago, IL, USA). Categorical variables were presented with numbers and percentages, while continuous variables were expressed as absolute numbers, mean, median and range. The Pearson Chi-square test was used to determine the comparability of categorical variables, whereas the Mann–Whitney U test was employed to evaluate the discrepancy between independent groups in numerical variables where the assumption of normality was not met. Univariable Cox regression analysis was used to calculate hazard ratios (HRs) and 95% confidence intervals (CIs) for factors affecting OS and PFS. Variables reaching statistical significance in univariable analysis were subsequently entered into multivariable Cox regression analysis using forward conditional selection to identify independent predictors of OS and PFS. Survival estimates were calculated using the Kaplan–Meier method, and differences between groups were compared using the log-rank test. Given the limited number of events (17 deaths and 25 recurrences), multivariable models were deliberately kept parsimonious. The events-per-variable ratio was monitored throughout model building; final models included no more than three independent predictors to minimize the risk of overfitting. These results should therefore be interpreted as exploratory. A two-sided *p*-value of <0.05 was considered statistically significant. Missing data were minimal in this cohort. Lymph node status was unavailable in 16 patients (17.0%) who did not undergo lymphadenectomy, and omental involvement status was unavailable in 27 patients (28.7%) who did not undergo omentectomy; these patients were excluded from analyses involving these specific variables. Complete data were available for all other variables, including p53 and MMR IHC status, tumor size, histologic grade, depth of myometrial invasion, and follow-up status. No imputation was performed.

## 3. Results

We enrolled patients diagnosed with LVSI-positive EEC who had surgery in the present research. Of the 94 patients with LVSI-positive tumors, 58 (61.7%) were classified as having focal LVSI and 11 (11.7%) as substantial LVSI, while 25 (26.5%) exhibited prominent LVSI. Table 1 shows detailed clinicopathologic features of all these patients in accordance with LVSI grading. The mean age at surgery was 59.28 ± 8.64 years (range, 27–82 years), and 79 patients (84.0%) were postmenopausal. The majority of tumors were FIGO grade 2 (*n* = 73, 77.7%), and deep myometrial invasion was present in 54 patients (57.4%); however, cervical stromal invasion was observed less frequently (*n* = 25, 26.6%).

Tumor size was significantly associated with LVSI extent (*p* = 0.003). A high proportion of patients with substantial and prominent LVSI had tumors measuring greater than 4 cm (63.6% and 60.0%, respectively) compared with those with focal LVSI (25.9%). Adnexal involvement was present in five patients (5.3%) and was significantly more common in the prominent LVSI group (16.0%) compared with focal (1.7%) and substantial (0.0%) LVSI (*p* = 0.021). Similarly, omental involvement was identified in 5 of 67 evaluable patients (7.5%) and was most frequent in patients with prominent LVSI (21.1%) compared with focal (2.6%) and substantial (0.0%) LVSI (*p* = 0.028). Lymphadenectomy was performed in 78 patients (83.0%). Among these, 11 (14.1%) had nodal metastases, and the rate of lymph node involvement increased significantly with LVSI extent: 6.5% in focal LVSI, 9.1% in substantial LVSI, and 33.3% in prominent LVSI (*p* = 0.012). Among the 94 patients, lymphadenectomy was omitted in 16 (17.0%) due to patient preference, medical comorbidities precluding extended surgery, or intraoperative findings indicating low-risk disease on frozen section analysis.

The surgical approach was determined by the attending surgeon. Seventy-one patients were operated via laparotomy, and 22 patients underwent laparoscopic approach, whereas robotic surgery was performed on only one patient. Pelvic washing for cytology was performed in 92.5% (n = 87) of cases, of which 1.1% (n = 1) was positive; this was in a patient with focal LVSI and was operated via laparotomy. A uterine manipulator was utilized in all laparoscopic procedures (*n* = 22). No significant association was observed between surgical approach and positive peritoneal cytology (*p* > 0.05).

With regard to immunohistochemistry-based surrogate molecular subclassification markers, 9.6% of tumors (*n* = 9) demonstrated a p53-abnormal staining pattern and 22.3% (*n* = 21) exhibited mismatch repair deficiency (MMRd). Complete IHC data for both markers were available for all 94 patients. No cases demonstrated overlap between p53-abnormal and MMRd status.

According to FIGO 2009 criteria, early-stage disease (stage I–II) was confirmed in 78 patients (83.0%). The detailed distribution was as follows: stage IA in 30 patients (31.9%), stage IB in 32 (34.0%), stage II in 16 (17.0%), stage IIIA in 2 (2.1%), stage IIIC1 in 9 (9.6%), and stage IVB in 5 (5.3%). A higher proportion of patients with prominent LVSI presented with stage III–IV disease (40.0%) compared with focal (8.6%) or substantial (9.1%) LVSI (*p* = 0.021).

When reclassified according to FIGO 2023 criteria, the stage distribution changed substantially (*p* < 0.001). Under this system, 38 patients (40.4%) were classified as stage I, 40 (42.6%) as stage II, 11 (11.7%) as stage III, and 5 (5.3%) as stage IV. All patients with focal LVSI who had myometrium-confined disease remained stage I (65.5% of focal LVSI cases), whereas all patients with substantial or prominent LVSI were reclassified to stage II or higher.

Adjuvant treatment was administered to 90 patients (95.7%). The distribution of treatment modalities across LVSI groups is detailed in Table 1. In the focal LVSI group, 18 patients (31.0%) received brachytherapy alone, 24 (41.4%) received EBRT alone, 5 (8.6%) received brachytherapy combined with EBRT, 7 (12.1%) received RT plus chemotherapy, and 4 (6.9%) declined adjuvant treatment. In the substantial LVSI group, two patients (18.2%) received brachytherapy alone, six (54.5%) received EBRT alone, two (18.2%) received combined brachytherapy and EBRT, and one (9.1%) received RT plus chemotherapy. In the prominent LVSI group, 7 patients (28.0%) received brachytherapy alone, 5 (20.0%) received EBRT alone, 3 (12.0%) received combined brachytherapy and EBRT, and 10 (40.0%) received RT plus chemotherapy. The addition of chemotherapy to RT was more common in patients with prominent LVSI compared with focal or substantial LVSI, reflecting the higher prevalence of advanced-stage disease in this group (*p* = 0.063).

The median time to recurrence was 63 (1–213) months. Among the 25 patients (26.6%) who experienced disease recurrence, 12.1% had focal LVSI, 27.3% had substantial LVSI, and 60.0% had prominent LVSI involvement (*p* < 0.001). We observed 6 (6.3%) locoregional and 19 (20.2%) distant recurrences. Tumor recurrence was reported in various locations, including the vaginal cuff, LN, the lung, the brain, the bone, the liver, the spleen and the peritoneum. Among those with occurred locoregional recurrence, 8.0% (2/25) had focal LVSI and 16.0% (4/25) had prominent LVSI. Of the 19 distant recurrences were observed, 13 of these were isolated: 16.0% (4/25), 12.0% (3/25), and 24.0% (6/25) in the focal, substantial and prominent LVSI groups, respectively; six of these were multifocal: only one had focal LVSI with MMRd and received adjuvant RT, while the remaining had prominent LVSI. Most of the distant recurrences were located at the lung (isolated). Not only the recurrence rate but also recurrence time was significantly correlated with prominent LVSI (median 27 months [1–97] for prominent LVSI, 48 months [3–167] for substantial LVSI, and 70.5 months [5–213] for focal LVSI). At the time of analysis, 17 patients (18.1%) had died, with mortality rates increasing progressively with LVSI extent: 5.2% in focal LVSI, 27.3% in substantial LVSI, and 44.0% in prominent LVSI (*p* < 0.001).

**Figure 3:**
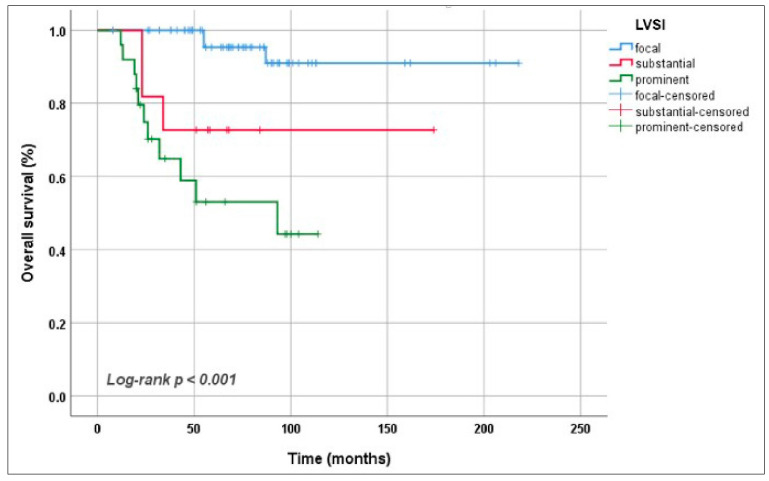
Overall survival according to the extent of lymphovascular space invasion.

**Figure 4:**
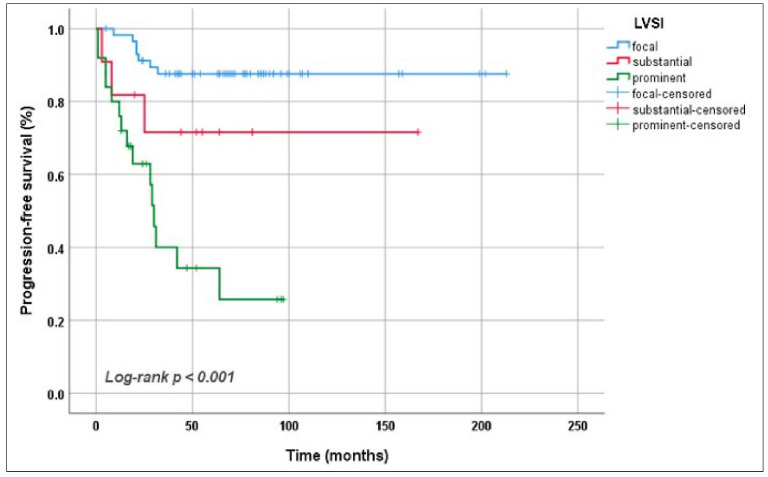
Progression-free survival according to the extent of lymphovascular space invasion.

The median follow-up duration was 67 (8–218) months. In the presence of prominent LVSI, it was observed that the 5-year OS rate dramatically decreased: 95.3%, 72.7% and 53.0% for focal, substantial and prominent LVSI, respectively (*p* < 0.001) (Figure 3). The same correlation was also found for PFS. The 5-year PFS rates were 34.3% for patients with prominent LVSI, 71.6% for those with substantial LVSI and 87.6% for those with focal LVSI (*p* < 0.001) (Figure 4).

According to multivariable Cox regression analysis results, high tumor size (HR: 10.185; 95% CI: 2.636–39.348; *p* = 0.001), prominent LVSI (HR: 14.309; 95% CI: 3.654–56.029; *p* < 0.001) and p53 abnormality (HR: 5.015; 95% CI: 1.622–15.506; *p* = 0.005) were independently associated with mortality. Other variables included in the multivariable analysis, namely, age (*p* = 0.059), FIGO 2023 stage II (*p* = 0.920), FIGO 2023 stage III (*p* = 0.186), FIGO 2023 stage IV (*p* = 0.190), substantial LVSI (*p* = 0.105), adnexal involvement (*p* = 0.571), lymph node involvement (*p* = 0.684), and omental involvement (*p* = 0.815), were found to be non-significant (Table 2).

According to multivariable Cox regression analysis results, high age (HR: 1.091; 95% CI: 1.041–1.144; *p* < 0.001), FIGO 2023 stage III (HR: 14.925; 95% CI: 2.369–94.014; *p* = 0.004), FIGO 2023 stage IV (HR: 17.593; 95% CI: 2.622–118.061; *p* = 0.003) and prominent LVSI (HR: 4.015; 95% CI: 1.410–11.428; *p* = 0.009) were independently associated with progression. Other variables included in the multivariable analysis, namely, FIGO 2023 stage II (*p* = 0.081), tumor size (*p* = 0.601), substantial LVSI (*p* = 0.516), myometrial invasion (*p* = 0.307), cervical stromal invasion (*p* = 0.988), adnexal involvement (*p* = 0.869), lymph node involvement (*p* = 0.106), omental involvement (*p* = 0.265) and p53 abnormality (*p* = 0.368), were found to be non-significant (Table 3).

## 4. Discussion

### 4.1. Summary of Main Results

Among 94 LVSI-positive EEC patients, 61.7% had focal, 11.7% substantial, and 26.6% prominent LVSI. Prominent LVSI was significantly associated with greater tumor size, nodal metastasis, advanced stage, and the highest recurrence rate. Five-year OS decreased stepwise from 95.3% in focal to 53.0% in prominent LVSI, and so did PFS, underscoring LVSI’s strong prognostic impact and highlighting LVSI extent as a key determinant of survival.

When analyzed according to the FIGO 2023 staging system—which incorporates substantial LVSI as a determinant of stage in early-stage disease—prominent LVSI retained independent prognostic significance on multivariable analysis for both OS and PFS. This finding suggests that stratification beyond the binary focal/substantial classification may provide additional prognostic information.

With respect to adjuvant treatment, the higher rate of combined radiotherapy and chemotherapy observed in the prominent LVSI group was largely a reflection of the greater prevalence of advanced-stage disease in this subgroup; treatment allocation was therefore driven primarily by disease burden rather than LVSI extent per se. The apparent absence of a survival benefit associated with the addition of chemotherapy to radiotherapy in this group most likely mirrors the unfavorable baseline characteristics of the patients who received it. Given the retrospective, non-randomized nature of treatment assignment, no inferences regarding adjuvant treatment efficacy can be drawn from these data.

### 4.2. Results in the Context of Published Studies

The presence of LVSI in EC has been identified as a well-documented risk factor associated with the development of LN metastasis, recurrence of disease, distant metastasis, and a decreased OS and PFS and is thus shown to play a pivotal role in guiding decisions regarding adjuvant treatment [15,24]. In the past few years, the assessment of LVSI has evolved from a mere reporting of presence or absence to a more detailed evaluation of its extent [15], and different scores have been proposed [5,12,13,25,26]. Quantifying the extent of LVSI has been proposed as a method of improving prognostication [7,27]. In this context, substantial LVSI (in opposition to focal or absent) has been shown to serve as a strong independent predictor of not only pelvic recurrence but also distal recurrence, and also OS [7,27].

A study by Bosse and colleagues [7] compared the extent of LVSI using two-, three-, and four-tiered scoring system for patients with early-stage EC. In their pooled PORTEC 1 and 2 analysis, substantial LVSI was clearly found as predictive risk factor for pelvic recurrence, with a 5-year risk of 15.3%, compared to those with focal LVSI (2.5%) and no LVSI (1.7%). Patients with substantial LVSI were shown to benefit from EBRT, resulting in a 5-year pelvic regional recurrence risk of 4.3%, compared to the 27.1% to 30.7% observed in those who did not receive adjuvant EBRT or brachytherapy [7,14]. In addition, with pelvic recurrence, substantial LVSI was shown to be an independent risk factor for distant recurrence and decreased OS. However, the PORTEC trials did not assess routine LNs; only LNs that were deemed to be suspect were removed [7,14].

There have been several studies that support the PORTEC trials. Relevant data from Italy evaluated patients with FIGO 2009 stage IA grade 1-2 EEC, using a cutoff of three or more vessels to define substantial LVSI, and 56.5% of patients underwent LN evaluation. The data showed that substantial LVSI was the strongest predictor of poor OS and an independent negative predictor of PFS and distant recurrence, despite those patients being more likely to receive adjuvant treatment [1,10]. Similarly, Restaino and colleagues [11] agreed that the effect of substantial LVSI dramatically decreased OS and PFS but testified that adjuvant RT did improve survival. The study also showed that distant recurrences were observed with greater frequency among patients with substantial LVSI [11]. Recently, the findings of a large study including patients with FIGO 2009 early-stage EC, 78.1% of whom underwent LN evaluation, indicated that the presence of substantial LVSI was associated with significantly worse prognostic outcomes. Conversely, the no and the focal LVSI exhibited similar prognostic outcomes, thereby indicating that they could be combined into one category [24]. In 2021, the Canada study investigated patients with FIGO 2009 early-stage EEC [1,27]. Substantial LVSI was defined as involving two or more vessels [1,27]. The results demonstrated that there was a relationship between substantial LVSI and pelvic and distant recurrence and PFS, but only 32% of patients underwent nodal assessment [1,27].

In other respects, other studies have shown disparate results. A multicenter retrospective cohort study published by Dagher and colleagues [6] investigated 1555 patients with pathology-proven node-negative FIGO 2009 stage I disease, using a three-tiered scoring system in accordance with the WHO definition. The 5-year PFS rates were as follows: 68.7% (substantial), 70.5% (focal) and 90.7% (no invasion) (*p* < 0.001); and the 5-year OS rates were 95.4% (no invasion), 82.2% (focal) and 76.5% (substantial) (*p* < 0.001) [6]. Unfortunately, the receipt of any adjuvant therapy was not supportive in terms of improving OS and PFS [6]. During the period of follow-up, Dagher and colleagues [1] observed that both focal and substantial LVSI were found to be a 3-fold increased risk of CI failure as opposed to no LVSI (19.5%, 19.0%, and 6.0% respectively). In contrast to the no LVSI, substantial LVSI was associated with an increased risk of distant recurrence [1]. The authors suggested that focal and substantial LVSI were associated with increased risk of disease progression and do not appear to be prognostically distinct; but focal and no LVSI have different prognostic outcomes and should not be combined into one category [6]. Several factors may account for the differences between our findings and those reported by Dagher and colleagues. First, our study exclusively enrolled LVSI-positive patients and compared outcomes across three LVSI tiers, whereas Dagher et al. included LVSI-negative cases as a reference and used a binary substantial/focal classification. Second, our cohort included patients across all FIGO stages, while their analysis was restricted to node-negative stage I disease, limiting direct comparability. Third, the higher rate of chemotherapy use in our prominent LVSI group (40.0% vs. 12.1% in focal LVSI) reflects the strong association between prominent LVSI and advanced-stage disease. These methodological differences highlight the need for standardized LVSI reporting criteria and prospective multi-institutional studies to clarify the independent prognostic contribution of LVSI extent.

Consistently, the publication by Bhatnagar and colleagues [28] identified patients with FIGO 2009 stage I and bilaterally pathology-proven node-negative EEC. To quantify LVSI, substantial LVSI was predicated on the PORTEC trials (three or more foci of LVSI) [28]. The data expressed that both focal and substantial LVSI had worse prognosis comparing with no LVSI, but there was no significantly distinction between focal and substantial LVSI for recurrence and survival [28]. On the other hand, Pifer and colleagues [14] evaluated 319 patients with FIGO stage I node-negative EEC, using three-tier LVSI scoring also based on the PORTEC trials, and reported no relationship between the extent of LVSI and recurrence patterns and 2-year survival rates; however, there was a marked discrepancy with higher administration of EBRT in patients with substantial LVSI (*p* < 0.001). The discrepancies among studies seeking out the prognostic importance of substantial LVSI lie in different cutoffs to define substantial LVSI, the classification of recurrence locations, nodal assessment, the rates of LVSI-positive patients and inter-observer variabilities in pathological evaluation.

### 4.3. Strengths and Weaknesses

To our knowledge, this is the first study investigating the only LVSI-positive patients classified by grading as focal, substantial, and prominent and comparing them with each other. In addition, all surgery was performed by gynecologic oncologists, and pathological sections were reviewed independently by gynecologic pathologists, which ensured the accuracy of the results. The majority of patients (83.0%) underwent LN assessment and except for four, all patients received adjuvant therapy. Finally, the data were well-documented with long and good follow-up periods, which provide reliable information about recurrence and survival. We acknowledge that our threshold of ten or more vessels for prominent LVSI is novel and requires external validation. This threshold was empirically derived to identify cases with exceptionally high tumor embolic burden. The substantial survival differences observed between the substantial (5–9 vessels) and prominent (≥10 vessels) groups—with five-year OS rates of 72.7% and 53.0%, respectively—suggest that this additional stratification captures clinically meaningful heterogeneity. We therefore caution against adopting this threshold in routine clinical practice pending independent replication. Future prospective, multi-institutional studies with predefined vessel-count cutoffs are needed to evaluate whether incorporating prominent LVSI into risk models improves prognostic discrimination beyond the standard focal/substantial classification.

Several limitations of this study must be acknowledged. First, the retrospective design and single-institution setting limit generalizability. Second, the relatively small sample size and limited number of events resulted in wide confidence intervals for some multivariable estimates. Third, POLE mutation testing was not performed; therefore, complete molecular classification according to The Cancer Genome Atlas or ProMisE frameworks could not be achieved. The p53 and MMR assessments represent IHC-based surrogates rather than comprehensive molecular profiling. Fourth, adjuvant therapy regimens and indications evolved over the study period, potentially introducing treatment heterogeneity. Given the limited number of events and the post hoc derivation of the cutoff, the multivariable estimates should be interpreted with caution, as the models may not be fully stable; independent replication in larger, prospectively designed cohorts is necessary.

A further interpretive consideration concerns the differential intensity of adjuvant treatment across LVSI groups. Patients with prominent LVSI received combined radiotherapy and chemotherapy substantially more often (40.0%) than those with focal LVSI (12.1%), largely reflecting their higher prevalence of advanced-stage disease. In this context, the independent prognostic effect of prominent LVSI on multivariable analysis—where FIGO 2023 stage was included as a covariate—suggests that its association with mortality and disease progression is not simply a proxy for advanced stage or more aggressive adjuvant treatment. Nonetheless, because treatment allocation was not randomized, residual confounding by indication cannot be excluded. The adverse prognosis associated with prominent LVSI persisted despite more intensive treatment, which, if anything, may lead to an underestimation of its true prognostic impact. Prospective studies with standardized treatment protocols will be necessary to disentangle the independent contribution of LVSI extent from treatment-related effects.

### 4.4. Implications for Practice and Future Research

It should be noted firstly that the heterogeneity inherent within the threshold definitions of substantial LVSI has a complicating effect upon the comparison of results across a range of different studies. Consistent with our findings, incorporating LVSI extent into risk stratification models—particularly identifying prominent LVSI—may improve prognostic accuracy and guide individualized treatment decisions. At this point, we believe that the LVSI scoring-system may be re-considered in an appropriate and meaningful manner for future aspects.

## 5. Conclusions

In summary, our findings demonstrate that further stratification of LVSI extent—specifically the identification of prominent LVSI—captures independent prognostic information not fully accounted for by FIGO 2023 staging alone, and that tumor size and p53 abnormality are additional determinants of outcome in LVSI-positive EEC. Prominent LVSI, in particular, identifies a group at highest risk of recurrence and poor survival. As these findings are derived from an exclusively LVSI-positive cohort and the prominent LVSI threshold was empirically defined, they should be considered exploratory. Future prospective, multi-institutional analyses with molecular information are warranted to further investigate the prognostic impact and treatment decisions associated with the extent of LVSI, which needs to be standardized globally.

## Figures and Tables

**Table 1:** Clinicopathologic features of all these patients in accordance with LVSI grading.

Patient Characteristic	N (%)	Focal LVSI, N (%)	Substantial LVSI, N (%)	Prominent LVSI, N (%)	
	Total = 94	Total = 58	Total = 11	Total = 25	*p*-Value
Age, years					0.769
Mean	59.28	59.62	57.55	59.24	
SD	8.64	8.89	8.45	8.38	
Menopausal status					0.535
Premenopausal	15 (16.0%)	8 (13.8%)	3 (27.3%)	4 (16.0%)	
Postmenopausal	79 (84.0%)	50 (86.2%)	8 (72.7%)	21 (84.0%)	
Grade					0.400
1	5 (5.3%)	4 (6.9%)	0 (0.0%)	1 (4.0%)	
2	73 (77.7%)	46 (79.3%)	7 (63.6%)	20 (80.0%)	
3	16 (17.0%)	8 (13.8%)	4 (36.4%)	4 (16.0%)	
Tumor size					**0.003**
≤4 cm	57 (60.6%)	43 (74.1%)	4 (36.4%)	10 (40.0%)	
>4 cm	37 (39.4%)	15 (25.9%)	7 (63.6%)	15 (60.0%)	
Myometrial invasion					0.225
<50%	40 (42.6%)	28 (48.3%)	5 (45.5%)	7 (28.0%)	
≥50%	54 (57.4%)	30 (51.7%)	6 (54.5%)	18 (72.0%)	
Cervical stromal invasion					0.258
Absent	69 (73.4%)	46 (79.3%)	7 (63.6%)	16 (64.0%)	
Present	25 (26.6%)	12 (20.7%)	4 (36.4%)	9 (36.0%)	
Adnexal involvement					**0.021**
Absent	89 (94.7%)	57 (98.3%)	11 (100.0%)	21 (84.0%)	
Present	5 (5.3%)	1 (1.7%)	0 (0.0%)	4 (16.0%)	
Lymph node involvement					**0.012**
Absent	67 (85.9%)	43 (93.5%)	10 (90.9%)	14 (66.7%)	
Present	11 (14.1%)	3 (6.5%)	1 (9.1%)	7 (33.3%)	
Omitted	16				
Omental involvement					**0.028**
Absent	62 (92.5%)	37 (97.4%)	10 (100.0%)	15 (78.9%)	
Present	5 (7.5%)	1 (2.6%)	0 (0.0%)	4 (21.1%)	
Omitted	27				
FIGO stage, 2009					0.104
IA	30 (31.9%)	21 (36.2%)	4 (36.4%)	5 (20.0%)	
IB	32 (34.0%)	21 (36.2%)	3 (27.3%)	8 (32.0%)	
II	16 (17.0%)	11 (19.0%)	3 (27.3%)	2 (8.0%)	
IIIA	2 (2.1%)	1 (1.7%)	0 (0.0%)	1 (4.0%)	
IIIB	0 (0.0%)	0 (0.0%)	0 (0.0%)	0 (0.0%)	
IIIC1	9 (9.6%)	3 (5.2%)	1 (9.1%)	5 (20.0%)	
IIIC2	0 (0.0%)	0 (0.0%)	0 (0.0%)	0 (0.0%)	
IVA	0 (0.0%)	0 (0.0%)	0 (0.0%)	0 (0.0%)	
IVB	5 (5.3%)	1 (1.7%)	0 (0.0%)	4 (16.0%)	
FIGO stage, 2009					**0.021**
I	62 (66.0%)	42 (72.4%)	7 (63.6%)	13 (52.0%)	
II	16 (17.0%)	11 (19.0%)	3 (27.3%)	2 (8.0%)	
III	11 (11.7%)	4 (6.9%)	1 (9.1%)	6 (24.0%)	
IV	5 (5.3%)	1 (1.7%)	0 (0.0%)	4 (16.0%)	
FIGO stage, 2023					**<0.001**
IA1	0 (0.0%)	0 (0.0%)	0 (0.0%)	0 (0.0%)	
IA2	17 (18.1%)	17 (29.3%)	0 (0.0%)	0 (0.0%)	
IA3	0 (0.0%)	0 (0.0%)	0 (0.0%)	0 (0.0%)	
IB	21 (22.3%)	21 (36.2%)	0 (0.0%)	0 (0.0%)	
IC	0 (0.0%)	0 (0.0%)	0 (0.0%)	0 (0.0%)	
IIA	9 (9.6%)	9 (15.5%)	0 (0.0%)	0 (0.0%)	
IIB	20 (21.3%)	0 (0.0%)	7 (63.6%)	13 (52.0%)	
IIC	11 (11.7%)	6 (10.3%)	3 (27.3%)	2 (8.0%)	
IIIA1	2 (2.1%)	1 (1.7%)	0 (0.0%)	1 (4.0%)	
IIIA2	0 (0.0%)	0 (0.0%)	0 (0.0%)	0 (0.0%)	
IIIB1	0 (0.0%)	0 (0.0%)	0 (0.0%)	0 (0.0%)	
IIIB2	0 (0.0%)	0 (0.0%)	0 (0.0%)	0 (0.0%)	
IIIC1	9 (9.6%)	3 (5.2%)	1 (9.1%)	5 (20.0%)	
IIIC2	0 (0.0%)	0 (0.0%)	0 (0.0%)	0 (0.0%)	
IVA	0 (0.0%)	0 (0.0%)	0 (0.0%)	0 (0.0%)	
IVB	5 (5.3%)	1 (1.7%)	0 (0.0%)	4 (16.0%)	
IVC	0 (0.0%)	0 (0.0%)	0 (0.0%)	0 (0.0%)	
FIGO stage, 2023					**<0.001**
I	38 (40.4%)	38 (65.5%)	0 (0.0%)	0 (0.0%)	
II	40 (42.6%)	15 (25.9%)	10 (90.9%)	15 (60.0%)	
III	11 (11.7%)	4 (6.9%)	1 (9.1%)	6 (24.0%)	
IV	5 (5.3%)	1 (1.7%)	0 (0.0%)	4 (16.0%)	
p53 abnormality					0.109
Absent	85 (90.4%)	55 (94.8%)	10 (90.9%)	20 (80.0%)	
Present	9 (9.6%)	3 (5.2%)	1 (9.1%)	5 (20.0%)	
MMR deficiency					0.928
Absent	73 (77.7%)	45 (77.6%)	9 (81.8%)	19 (76.0%)	
Present	21 (22.3%)	13 (22.4%)	2 (18.2%)	6 (24.0%)	
Adjuvant therapy					0.063
Absent	4 (4.3%)	4 (6.9%)	0 (0.0%)	0 (0.0%)	
Brachytherapy	27 (28.7%)	18 (31.0%)	2 (18.2%)	7 (28.0%)	
EBRT	35 (37.2%)	24 (41.4%)	6 (54.5%)	5 (20.0%)	
Brachytherapy + EBRT	10 (10.6%)	5 (8.6%)	2 (18.2%)	3 (12.0%)	
RT + CT	18 (19.1%)	7 (12.1%)	1 (9.1%)	10 (40.0%)	
Relapse					**<0.001**
Absent	69 (73.4%)	51 (87.9%)	8 (72.7%)	10 (40.0%)	
Present	25 (26.6%)	7 (12.1%)	3 (27.3%)	15 (60.0%)	
Exitus					**<0.001**
Absent	77 (81.9%)	55 (94.8%)	8 (72.7%)	14 (56.0%)	
Present	17 (18.1%)	3 (5.2%)	3 (27.3%)	11 (44.0%)	

SD: standard deviation; LVSI: lymphovascular space invasion; MMR: mismatch repair; RT: radiotherapy; CT: chemotherapy. Bold *p*-values indicate statistical significance (*p* < 0.05).

**Table 2:** Cox regression analysis for predictors of survival outcome.

	Univariable			Multivariable		
Variables	HR	95% CI	*p*-Value	HR	95% CI	*p*-Value
Age, years	1.061	1.002–1.122	**0.042**			0.059
Menopausal status						
Premenopausal	1					
Postmenopausal	27.130	0.118–62.223	0.234			
FIGO stage, 2009						
I	1					
II	1.053	0.223–4.964	0.948			
III	2.473	0.655–9.329	0.181			
IV	9.911	2.954–33.252	**<0.001**			
FIGO stage, 2023						
I	1					
II	4.562	0.967–21.537	0.055			0.920
III	6.528	1.089–39.123	**0.040**			0.186
IV	26.331	4.774–145.233	**<0.001**			0.190
Grade						
1	1					
2	0.313	0.068–1.452	0.138			
3	1.149	0.230–5.742	0.866			
Tumor size						
≤4 cm	1			1		
>4 cm	6.916	2.240–21.350	**0.001**	10.185	2.636–39.348	**0.001**
LVSI						
Focal	1			1		
Substantial	6.275	1.260–31.260	**0.025**	3.807	0.756–19.165	0.105
Prominent	12.937	3.587–46.662	**<0.001**	14.309	3.654–56.029	**<0.001**
Myometrial invasion						
<50%	1					
≥50%	1.633	0.599–4.448	0.338			
Cervical stromal invasion						
Absent	1					
Present	2.423	0.918–6.393	0.074			
Adnexal involvement						
Absent	1					
Present	4.534	1.293–15.900	**0.018**			0.571
Lymph node involvement						
Absent	1					
Present	5.096	1.691–15.360	**0.004**			0.684
Omental involvement						
Absent	1					
Present	7.731	2.357–25.356	**0.001**			0.815
p53 abnormality						
Absent	1					
Present	4.725	1.661–13.436	**0.004**	5.015	1.622–15.506	**0.005**
MMR deficiency						
Absent	1					
Present	2.368	0.874–6.420	0.090			
Adjuvant therapy						
Absent	1					
RT	0.536	0.067–4.289	0.556			
RT + CT	2.539	0.316–20.388	0.381			

HR: hazard ratio; CI: confidence interval; LVSI: lymphovascular space invasion; MMR: mismatch repair; RT: radiotherapy; CT: chemotherapy. Multivariable analysis was performed using the forward conditional selection method. “FIGO stage, 2009” was omitted from multivariable analysis to avoid multicollinearity with “FIGO stage, 2023” (r = 0.770; *p* < 0.001). Given the limited number of events and the exploratory model-building approach, these multivariable estimates should be interpreted with appropriate caution. Bold *p*-values indicate statistical significance (*p* < 0.05).

**Table 3:** Cox regression analysis for predictors of progression outcome.

	Univariable			Multivariable		
Variables	HR	95% CI	*p*-Value	HR	95% CI	*p*-Value
Age, years	1.058	1.012–1.105	**0.012**	1.091	1.041–1.144	**<0.001**
Menopausal status						
Premenopausal	1					
Postmenopausal	2.596	0.612–11.021	0.196			
FIGO stage, 2009						
I	1					
II	2.317	0.776–6.922	0.132			
III	4.531	1.612–12.738	**0.004**			
IV	14.778	4.757–45.908	**<0.001**			
FIGO stage, 2023						
I	1					
II	7.243	1.616–32.457	**0.010**	4.501	0.829–24.429	0.081
III	13.854	2.792–68.740	**0.001**	14.925	2.369–94.014	**0.004**
IV	46.333	8.680–247.327	**<0.001**	17.593	2.622–118.061	**0.003**
Grade						
1	1					
2	1.263	0.169–9.442	0.820			
3	1.712	0.200–14.682	0.624			
Tumor size						
≤4 cm	1					
>4 cm	3.693	1.626–8.390	**0.002**			0.601
LVSI						
Focal	1					
Substantial	2.774	0.717–10.734	0.140	1.618	0.379–6.918	0.516
Prominent	8.275	3.335–20.532	**<0.001**	4.015	1.410–11.428	**0.009**
Myometrial invasion						
<50%	1					
≥50%	3.439	1.289–9.171	**0.014**			0.307
Cervical stromal invasion						
Absent	1					
Present	2.575	1.167–5.683	**0.019**			0.988
Adnexal involvement						
Absent	1					
Present	4.228	1.448–12.348	**0.008**			0.869
Lymph node involvement						
Absent	1					
Present	4.783	1.900–12.044	**0.001**			0.106
Omental involvement						
Absent	1					
Present	8.005	2.807–22.826	**<0.001**			0.265
p53 abnormality						
Absent	1					
Present	4.494	1.873–10.784	**0.001**			0.368
MMR deficiency						
Absent	1					
Present	1.638	0.682–3.933	0.270			

HR: hazard ratio; CI: confidence interval; LVSI: lymphovascular space invasion; MMR: mismatch repair. Multivariable analysis was performed using the forward conditional selection method. “FIGO stage, 2009” was omitted from multivariable analysis to avoid multicollinearity with “FIGO stage, 2023” (r = 0.770; *p* < 0.001). As with the OS analysis, the relatively small number of recurrence events limits the precision of these multivariable estimates, and wide confidence intervals for some covariates reflect this uncertainty. Bold *p*-values indicate statistical significance (*p* < 0.05).

## Data Availability

Due to hospital policies, patient data and study materials cannot be shared. However, the data are available from the corresponding author upon reasonable request.

## Abbreviations

BSO	Bilateral Salpingo-Oophorectomy
BPPALND	Bilateral Pelvic ± Para-Aortic Lymph Node Dissection
CA-125	Cancer Antigen 125
CI	Confidence Interval
CT	Chemotherapy
EBRT	External Beam Radiation Therapy
EC	Endometrial Cancer
ECG	Electrocardiogram
EEC	Endometrioid Endometrial Cancer
ESGO	European Society of Gynaecological Oncology
ESP	European Society of Pathology
ESTRO	European Society for Radiotherapy and Oncology
FIGO	International Federation of Gynecology and Obstetrics
H&E	Hematoxylin and Eosin
HR	Hazard Ratio
LN	Lymph Node
LVSI	Lymphovascular Space Invasion
L/S	Laparoscopy
L/T	Laparotomy
MELF	Microcystic, Elongated and Fragmented Pattern
MI	Myometrial Invasion
MMRd	Mismatch Repair Deficiency
MRI	Magnetic Resonance Imaging
NACT	Neoadjuvant Chemotherapy
NCCN	National Comprehensive Cancer Network
OS	Overall Survival
PALND	Para-Aortic Lymph Node Dissection
PET-CT	Positron Emission Tomography/Computed Tomography
PFS	Progression-Free Survival
PLND	Pelvic Lymph Node Dissection
PORTEC	Post-Operative Radiation Therapy in Endometrial Carcinoma
RT	Radiotherapy
SPSS	Statistical Package for the Social Sciences
TVUSG	Transvaginal Ultrasonography
WHO	World Health Organization
